# The Use of Botulinum Toxin in Complex Hernia Surgery: Achieving a Sense of Closure

**DOI:** 10.3389/fsurg.2021.753889

**Published:** 2021-10-01

**Authors:** Thomas Whitehead-Clarke, Alastair Windsor

**Affiliations:** ^1^Centre for 3D Models of Health and Disease, Division of Surgery and Interventional Science, University College London, London, United Kingdom; ^2^Alastair Windsor, Princess Grace Hospital, HCA Healthcare, London, United Kingdom

**Keywords:** Botox, Botulinum, complex, abdominal wall, hernia

## Abstract

Abdominal wall surgeons have developed a host of tools to help facilitate fascial closure. Botulinum toxin A is one of the most recently identified treatments and has grown in popularity over recent years; showing great promise in a number of case series and cohort studies. The toxin paralyses lateral abdominal wall muscles in order to increase laxity of the tissues—facilitating medialisation of the rectus muscles. Several research groups around the world are developing expertise with its use-uncovering its potential. We present a review of the relevant literature over the last two decades, summarising the key evidence behind its indications, dosing and effects.

## Introduction

As abdominal wall surgery evolves, the increasing challenges posed by large, complex hernias continue to require novel interventions. Nowhere is this more clearly demonstrated than the challenge of achieving fascial closure in hernias with large defects or significant loss of domain (LOD). Techniques such as Ramirez' components separation (CS) and Progressive Preoperative Pneumoperitoneum (PPP) have been described in the literature for decades (1, 2) and are still mainstays in complex hernia management. In recent years, the use of Botulinum toxin A (BTX) has grown in popularity and now represents an emerging frontline technique to be used either in conjunction with (3) or instead of (4) other established practises.

BTX is a neurotoxin that acts selectively on presynaptic cholinergic nerve terminals, blocking the release of acetylcholine resulting in temporary flaccid muscle paralysis without systemic effects. Applying this neurotoxin to the lateral muscle complex (Internal oblique, External oblique and Transversus Abdominus) can paralyse and subsequently elongate the muscle complex; facilitating medialisation of the rectus muscles and abdominal wall closure. Given its relative non-invasiveness, it represents a useful adjunct to abdominal wall surgery that a number of groups have now been exploring for several years. We review the last 20 years of BTX literature and explore its development, indications, effects, and future.

## Early Development

The use of BTX to treat herniation can be found in the literature as far back as 1999 when it was used to treat muscular hernias of the leg (5). It was in 2006 when Cakmak et al. investigated its use *in vivo* with a study of fifteen rats (6); identifying its potential to facilitate abdominal wall closure. Following this work, a number of small observational studies brought BTX into the surgical mainstream. In 2009, a prospective review of 12 patients by Ibarra-Hurtado et al. was the first to investigate BTX as an adjunct for hernia repair (7). With their regime of 5 injections into the lateral abdominal wall, they demonstrated a statistically significant reduction in fascial defect width (mean 5.25 cm) at around 4 weeks after treatment. Half of patients studied (mean defect width 13.85 cm) needed no further CS to achieve fascial closure.

Years later, a Mayo Clinic group produced two articles that described BTX use slightly differently. In 2011 Smoot et al. (8) published a case report exploring BTX's use for analgesic purposes; describing the successful treatment of patient reported muscle spasms after laparoscopic hernia surgery. Soon afterwards, the same group (Zielinkski et al.) documented their use of BTX to facilitate fascial closure in patients treated with an open abdomen. In their work, Zielinski et al. achieved an 83% rate of fascial closure, which increased to 89% for patients given BTX within 24 h of their index laparostomy (9). Further studies in 2014 (10, 11) added to the evidence base for BTX as a potentially useful tool to help abdominal wall closure.

## Indications and Use With PPP

Whilst studies have shown early promise regarding BTX and its efficacy, the hernia community are yet to agree on precise indications for its use. Unlike CS, BTX must be administered weeks in advance of surgery and involves a significant financial cost, making preoperative patient selection critical. A number of groups have described potential indications for BTX which range from specific hernia characteristics such as defect width, to less specific criteria driven by expert opinion or multi-disciplinary team meetings. A systematic review by van Rooijen et al. (12) concluded that whilst certain given indications for PPP were fairly definitive (e.g., loss of domain more than 20%), indications for BTX were less so.

When Nielsen et al. looked at the long-term safety of BTX, it was given on “*clinical suspicion of difficult closure*,” with additional CS if necessary (13). Similarly, when Deerenberg produced a prospective study of 108 patients from the Carolinas Medical Centre, the decision to use BTX was based on opinion and discussion among expert hernia surgeons (14). Others have identified further qualitative CT findings such as shortened or thickened lateral abdominal muscles (12).

Others have described more quantitative indications for BTX use—such as Catalan-Garza et al. who treated 36 patients with BTX—all with hernias wider than 100 mm or with 20% LOD (15). In their prospective study of 56 patients, Rodriguez-Acevedo et al. discussed BTX in patients with “*complex ventral hernias*” (16). These hernias were defined as recurrent or traumatic hernias with a defect of more than 6 cm and/or a loss of domain >20% (16); a definition they appear to alter slightly in later work (17).

Several groups with extensive BTX experience have developed specific treatment algorithms, describing precise indications (17–19). These algorithms often discuss BTX use in association with PPP, describing their separate indications and potential synergy—PPP potentially helping to stretch out pre-paralysed abdominal wall muscles (17).

The Spanish group led by Bueno-Lledó first described their algorithm for the combined use of BTX and PPP in 2017 (3). Their most recent work summarises their first 100 patients treated with this regimen—outlining their newest algorithm (19). This group uses BTX with transverse fascial defects of 12 cm or greater, and PPP treatment for any hernia with LOD >20% Tanaka et al. (20) irrespective of defect size.

Yurtkap et al. describe another algorithm where BTX is given for facial defects 14–18 cm in size (18). If 18–22 cm in size, PPP is added for hernias with significant LOD; both PPP and BTX are used for fascial defects >22 cm. They also describe omitting BTX in cases without a hypercontracted lateral muscle complex (18).

The Australian study by Jacombs et al. (17) also shared their BTX algorithm, where it is given with any fascial defect over 5 cm in width. PPP is added with fascial defects ≥15 cm, infraumbilical defects ≥9 cm or LOD ≥20%. Their study suggested that in many cases, BTX represents the only technique required to close facial defects <12 cm in width (76.7% of these cases) (17). This work suggests a paradigm shift in the use of BTX; presenting it as the primary method to assist fascial closure, as opposed to other algorithms which use CS as a first-line technique (18). This thinking was adopted by Bueno-Ledó et al. in their 2020 paper, investigating whether certain hernias could be successfully “*downstaged*” with BTX to avoid CS (4). The study prospectively followed 80 patients with hernia defects 11–17 cm in width. Forty patients underwent each treatment arm (BTX vs. CX) and were followed up for 2 years. They identified not only that certain hernias could be successfully downstaged to avoid CS, but there was also a significant increase in surgical site occurrences (SSOs) associated with the CS group (4). Whilst BTX may have the potential to prevent CS and therefore the potential risk of some SSOs, there is insufficient data regarding the cost/benefit analysis of use; especially given its significant financial burden.

Some studies have tried to tease apart the relative benefits of PPP and BTX in terms of assisting fascial closure. Some have suggested that PPP may offer no additional lateral abdominal wall length when used with BTX (16). Another study set out to establish the benefits of BTX + PPP treatment (*n* = 13) as opposed to PPP only (*n* = 28). They concluded that whilst the addition of BTX provided no additional abdominal volume, it helped reduce the incidence of SSOs (21).

## Treatment Protocols—Dosing, Timing, and Injection Sites

Due to its relative novelty, the ideal technique for BTX injection is not yet a matter of consensus (22), and insufficient evidence exists that one particular injection protocol is superior (15). BTX injection technique is multifaceted, including dosing, timing, injection sites, and adjunctive techniques.

### Dosage

Appropriate dosage for BTX use in the abdominal wall has been described anywhere between 100 IU (10) and 500 IU (3, 4, 19). The doses most frequently encountered in the literature being 200 IU (23), 300 IU (8, 24), and 500 IU (19). Some small studies have also given different doses to each patient throughout the study (25). The total dose is then divided equally between 3 and 5 injection sites each side of the midline. Some groups have altered their practise over the last decade; such as Jacombs et al. (17) who describe their reduction from 300 IU between 2012 and 2016 to 200 IU between 2016 and 2019. This reduction was related to a reduction from infiltrating 3 layers of the lateral muscle complex to just 2 layers.

Some discrepancy in dosage between groups may be due to different BTX preparations, such as Botox® or Dysport®. In their largest, most recent paper Bueno Lledó et al. (19) describe using Dysport® (Ipsen, Boulogne-Billancourt, France) whereas both Yurtkap et al. (18) and Deerenberg et al. (14) describe using Botox (Botulinum toxin Allergan, Inc., Irvine, California). Jacombs et al. (17) describe using an equivalent dose of either of the two formulations. Recent literature has described a possible 3:1 concentration ratio between the two formulations; (26) a factor clinicians should be aware of when choosing a treatment regime.

### Injection Sites

The technique originally described by Ibarra-Hurtado et al. involved 500 IU injected over 5 sites on each side: “*two over the midaxillary line, between the costal border and the superior iliac crest, and three over the external oblique muscle”* (7). Years later, when Smoot et al. published their experience using BTX for post-op analgesia they described a 3-site, 300 IU technique (8) without specifying the injection sites. This method was then better described in two publications by the same group, describing six injection sites in total: “*right/left subcostal; right/left anterior axillary; right/left lower quadrants*” (9, 27).

Some have suggested that nearly all published BTX injection protocols adhere to one of these two camps (Ibarra-Hurtado OR Zielinski/Smoot) (18). Groups like the Spanish Bueno-Lledó group have followed the description by Ibarra-Hurtado et al. giving BTX in 5 very similar locations to the Mexican group (3). Others such as Yurtkap et al. and Catalan-Garcia et al. have adopted the Zielinski/ Smoot protocol (15, 18). Some studies have used mixed protocols, using the higher 500 IU dose but between 3 injection sites (21).

When BTX use was first described, convention was that all three (External Oblique, Internal Oblique and Transversus Abdominus) lateral abdominal wall muscles were infiltrated. This idea has however been challenged in recent years. In their 2020 paper, Elstner et al. conducted a prospective study of 46 patients to assess whether selective 200 IU BTX infiltration of only the 2 most superficial lateral muscle layers could achieve a sufficient medialisation of the rectus muscles (23). Their conclusion that this technique was effective, altered their practise for some patients. This new 200 IU regime was used in patients with certain back complaints, due to Tranversus Abdominis' key role in thoracic spine stabilisation. This practise has been adopted by other groups (14) who noted no difference in numbers of patients requiring fascial release between 2 and 3 layer infiltration.

Other groups have made use of certain adjuncts to aid the BTX infiltration process. The Bueno-Lledó group routinely use electromyography (EMG) to ensure that the BTX is injected where it is most effective (19). They argue that this establishes whether the targeted muscle is denervated or fibrotic (28).

### Timing

Due to its long-acting effects, the ideal timing of pre-operative BTX likely involves a window of several days and has been described differently between authors. Early papers affirmed that the maximum effect of BTX is achieved at 2 weeks after injection (9, 27), however a recent systematic review and meta-analysis found BTX being given anywhere between 6 and 45 days before surgery (12). When originally described, CT images were reviewed 4 weeks post BTX—upon which most patients has experienced a reduction in fascial defect size (7). The Bueno-Lledó group aim to give BTX 4 weeks before surgery and have a mean pre-op injection of around 32–38 days (3, 19). This is similar to Deerenberg et al. who gave BTX an average of 32.5 days pre-operatively (14). Jacombs et al. give BTX 7–30 days pre-operatively, unless the hernia is particularly large in which case BTX is given between 21 and 30 days (17). In their 2021 paper Yurtkap et al. gave BTX a median of 45 days before surgery but with a large range of anywhere between 28 and 119 days (18). Please see Table 1 for a summary of recent studies and their BTX treatment regimes.

**Table 1:** BTX dosing at a glance; a list of recently published articles on BTX use and their treatment regimes.

**References**	**Patient cohort**	**Dosing**	**Injection sites**	**Pre-op timing**	**Formulation**
Jacombs et al. (17)	*n* = 88	200 or 300 units	3 sites bilaterally	7–30 days	Botox®/Dysport®
Yurtkap et al. (18)	*n* = 23	300 units	3 sites bilaterally−3 layers	Median 45 days (28-119).	Botox®
Deerenberg et al. (14)	*n* = 118	300 units (200 units for 2 layers)	3 sites bilaterally−2 or 3 layers	Mean 32.5 days.	Botox®
Bueno-Lledo et al. (19)	*n* = 100	500 units	5 sites bilaterally−3 layers	Mean 38.3 days (33-48).	Dysport®

## Clinical Benefits of BTX Use

### Muscular Paralysis and Fascial Closure

The precise quantifiable benefits of BTX treatment are yet to be established. One issue complicating the evidence is the number of different criteria used to quantify BTX's effects, creating heterogeneity. Commonly recorded outcomes such as rate of fascial closure can be confounded by various factors, making other measures such as reduction in fascial defect or increase in lateral muscle length more objective and repeatable outcomes.

When first described, the efficacy of BTX was assessed by its capacity to reduce hernia defect size (7). Ibarra-Hurtado described 10 patients in which the mean hernia defect had been reduced by a mean of 5.25 cm from an original mean defect size of 13.85 cm (7). Chavez-Tostado et al. also analysed reduction in hernia defect width after only 100 IU of BTX but found no significant improvement (10).

Five years after their initial publication, Ibarra-Hurtado et al. published the results for 17 of their BTX patients – this time describing the effects of BTX upon lateral muscle elongation. They concluded that the BTX treatment facilitated a reduction in lateral muscle thickness by around a 1 cm, and an increase in lateral muscle length by 2.44 and 2.59 cm on the left and right, respectively (11). Hernia Institute Australia have published their outcomes in terms of lateral muscle elongation on several occasions. They have previously published mean increases of 4.2 (16) and 4 cm (29), and their largest cohort to date achieved a mean increase in lateral length of 4.7 cm per side (17).

There have now been several systematic reviews that have looked at the quantifiable benefits of BTX. Early reviews only assessed fascial closure rates and hernia defect reduction (30) whereas others chose to focus on fascial closure rates as a primary variable (12). Wegdam et al. (31) focused specifically on the quantifiable benefits of preoperative BTX. Their intended primary outcome was the elongation of the lateral muscle complex and secondarily reviewed the reduction in fascial defect width. They analysed 14 studies which summarised the findings for 344 patients. They identified four papers that had measured lateral muscle complex elongation — giving a median elongation of around 4 cm. This result was similar to a previous systematic review (3.33 cm) by Weissler et al. (32). When the review looked at their secondary effects such as reduction of hernia defect width, they identify three studies that reveal a defect reduction of 4.8 cm, and two other studies that reveal a statistically significant reduction of 5.1 cm (31). In the meta-analysis from Weissler et al. a mean hernia width reduction was identified at 5.79 cm (32).

Some studies have focused on measuring the potential improvements to LOD measurements. Bueno-Lledó et al. observed a 15% reduction in the Hernia/Abdomen volume ratio (VIH/VAC) when using both BTX and PPP (19). In an earlier study focusing on 70 patients with LOD hernias (28) there was a similar mean reduction in the VIH/VAC ratio of 16.6%.

### Post-operative Analgesia

BTX was originally investigated for managing post-operative abdominal wall pain in 2011 when Smoot et al. gave BTX injections to treat muscular spasms after a laparoscopic hernia repair. They achieved a reduction in pain scores which lasted through 3 months of follow-up (8). Zendejas et al. conducted a larger study where they compared the post-op pain scores and opioid use in 22 BTX cases and 66 controls. They were able to conclude that in at least 2 of the 6 post-operative days assessed, that BTX patients were taking a significantly smaller dose of opiate analgesia, and also reported less pain on post op days 2 and 4 (27). There is not complete consensus however—one study of 97 BTX patients noticed they experienced more pain at 30 days post-op than the non BTX group (*n* = 291) (22).

### Prevention of Recurrence

Whilst the literature lacks robust evidence, some have asserted that BTX treatment may help reduce hernia recurrence. Theoretically the paralysis of the lateral abdominal wall muscles would reduce tension across the wound, creating a more tension-free healing process. van Rooijen et al. (12) and Deerenberg et al. (14) have both commented on the particularly low hernia recurrence rates after BTX treatment, the latter adding that insufficient long term studies exist to confirm how BTX may effect recurrence rates. Rodiriguez-Acevedo et al. assert in their work that that BTX helps to protect midline wounds over the initial 3 months of healing (16). The same group have also described giving BTX post-operatively as a prophylactic adjunct to aid the initial healing of the rectus sheath (33).

### Complications and Patient Experience

After a decade of use, BTX seemingly offers a low side-effect profile, and has been described as so by analysis from the Abdominal Core Health Quality Collaborative (22). Its use has been described as either safe, low morbidity or lacking complications in several publications (13–15, 22). Given its inherent non-invasiveness, it is likely to be significantly safer than techniques such as PPP. Yurtkap et al. found that nearly one third of their 17 PPP patients had suffered complications, without any of their BTX patients suffering similarly (18). This is not to say that BTX is without side-effects, and those commonly described in the literature include weak cough, back pain, and superficial bruising at the site of injection (12). Some efforts have been made to mitigate these side-effects, such as Jacombs et al. providing their BTX patients with abdominal binders after treatment (17). The same group from Hernia Institute Australia have also begun omitting injections into the Transversus Abdominis for patients with back issues (23).

## Discussion

After over a decade of use, BTX treatment has displayed a great potential as an adjunct for the closure of complex abdominal wall hernias. Whilst treatment protocols differ, 200–500 IU given between 6 and 10 sites around 30 days preoperatively appears to facilitate measurable results. It is a relatively safe technique with only minimal side-effects and may offer as much as 4 cm lateral muscle elongation per side if done effectively.

Authors continue to describe BTX use for various other general surgical procedures. A number of authors have documented their successful use of BTX to help with the repair of challenging inguino-scrotal hernias (34–37), as well as abdominal wall defects in children (38, 39) and in the practise of giant hiatus hernia repair (40).

Perhaps the area of greatest potential for future BTX use is the “downgrading” of complex hernias - avoiding the need for CS. This development could significantly shorten operating times and avoid potential wound complications associated with extensive soft tissue dissection. Abdominal wall wound complications are known to carry a significant morbidity; increasing healthcare costs and length of stay for patients. Another theoretical benefit of BTX may be its potential to reduce muscle tone post-operatively, decreasing wound tension, and improving recurrence rates. More long-term trials are required to develop strong evidence for BTX's effects, but its cost may currently act as a barrier to broader take-up. Greater dialogue between surgeon groups, insurance companies, and industry may increase its use—producing larger, more robust data sets. Only then will we be in a position to identify its potential to reduce costs and improve patient outcomes long-term.

## Author Contributions

TW-C developed the manuscript. AW reviewed and edited the manuscript. TW-C and AW are responsible for its content.

## Conflict of Interest

AW interest statement: Allergan - Research Grant TelaBio, Medtronic - Speaker Bureau. The remaining author declares that the research was conducted in the absence of any commercial or financial relationships that could be construed as a potential conflict of interest.

## Publisher's Note

All claims expressed in this article are solely those of the authors and do not necessarily represent those of their affiliated organizations, or those of the publisher, the editors and the reviewers. Any product that may be evaluated in this article, or claim that may be made by its manufacturer, is not guaranteed or endorsed by the publisher.
